# Association of Internet use disorder and the use of traditional watches

**DOI:** 10.1016/j.abrep.2026.100685

**Published:** 2026-02-28

**Authors:** Lea-Christin Wickord, Anja Bischof, Christopher Kannen, Dimitri Löchner, Harriet Salbach, Thomas Mößle, Lara Zumrode, Klaus Wölfling, Hans-Jürgen Rumpf, Christian Montag

**Affiliations:** a Institute of Psychology, University Koblenz, Universitätsstr. 1, Koblenz 56070, Germany; b Department of Psychiatry and Psychotherapy, University of Lübeck, Ratzeburger Allee 160, Lübeck 23538, Germany; c Institute of Psychology and Education, Ulm University, Helmholtzstraße 8/1, Ulm 89081, Germany; d Institute of Psychology, University of Tartu, Näituse 2, Tartu 50409, Estonia; e Department of Education and Psychology, Freie Universität Berlin, Habelschwerdter Allee 45, Berlin 14195, Germany; f Media Protect e.V., Schlosserstr. 7/1, Emmendingen 79312, Germany; g State Police College of Baden-Württemberg, Sturmbühlstr. 250, Villingen-Schwenningen 78054, Germany; h Outpatient Clinic for Behavioral Addictions, University Medical Center Mainz, Untere Zahlbacher Str. 8, Mainz 55131, Germany; i Centre for Cognitive and Brain Sciences, Institute of Collaborative Innovation, University of Macau, Avenida da Universidade, Macau SAR, China; j Department of Psychology, Faculty of Social Sciences, University of Macau, Avenida da Universidade, Macau SAR, China; k Department of Computer and Information Science, Faculty of Science and Technology, University of Macau, Avenida da Universidade, Macau SAR, China

**Keywords:** Timekeeper, Watches, Alarm clocks, Structuring everyday life, Internet use disorder

## Abstract

This study examines the association between the use of so-called “timekeepers”, such as traditional wristwatches or classic alarm clocks, or digital “timekeepers” as smartphones or smartwatches, and Internet use disorder. Based on theoretical models it was examined whether certain everyday habits are related to the extent of problematic online behavior. In a cross-sectional online survey (N = 6,615), participants answered the Compulsive Internet Use Scale (CIUS) and questions about their usage behavior in the context of smartphones, wrist watches, and alarm clocks.

Participants who stated that their Internet use was excessive or that they stayed online longer than planned after looking at the clock function on their smartphone had significantly higher CIUS scores. The same was true for people who did not wear a watch, used their smartphone as an alarm clock, took it into the bedroom, or went online immediately after waking up. The time of first smartphone use in the morning proved to be a significant predictor of problematic use, whereas the last time using the smartphone before going to bed was not.

The findings replicate and extend previous studies and suggest that non-Internet-connected timekeepers are associated with less dysfunctional Internet and smartphone usage patterns. The use of the smartphone as a multifunctional device could be suggested as a potential trigger for unwanted media use. However, targeted everyday modifications − such as the use of classic watches − might contribute to the prevention of Internet use disorder which needs to be tested in future studies.

## Highlights

• Subjective overuse and post-time-check use strongly predict Internet use disorder.
• Analog timekeepers (watches / clocks) link to less dysfunctional smartphone usage.
• Less problematic internet use found for analog watches than smartwatches.
• First daily smartphone use time predicts problematic use severity.

## 1 Introduction

Internet-enabled devices such as computers, tablets, and smartphones are now widespread in our society (Statista., 2024). Smartphones in particular are constant companions in everyday life and enable permanent access to the Internet. Smartphones are no longer only portable phones but are used for listening to music, as a camera, as a navigation device, or for reading news and surfing the Internet. Smartphones have increasingly replaced devices that specialize in these functions. The presence of smartphones and the associated easier access to social media and other Internet applications offer many advantages, but can also become detrimental if use becomes excessive (Elhai et al., 2017, Kuss and Pontes, 2019).

Excessive and problematic use is particularly evident in connection with the smartphone, as it combines many usage options in one device; e.g. access to news and information from the Internet, communication with social contacts, and life organization and can satisfy many different motives at the same time (Wickord & Quaiser-Pohl, 2023). The motives that increase Internet and smartphone use include the need for information (orientation seeking, advice, learning), the need for entertainment (escapism, relaxation, sexual stimulation), the need for personal identity (search for models of behavior, reinforcement of personal values) and the need for integration and social interaction (substitute for sociability, role model, conversation) (Mangold et al., 2004). Due to the increasing integration of different services within the smartphone, the duration of use is increasing heavily (Panova & Carbonell, 2018). This is also reflected in the uses-and-gratifications approach, a model of media use research, which focuses on gratifications (satisfaction of needs). The model describes that users devote themselves to certain media offerings to derive certain benefits from the respective media (Whiting & Williams, 2013). As a further development of the uses and gratifications approach, Compensatory Internet Use Theory (CIUT) attempts to explain that media is consumed in order to forget negative life events and stress factors, which leads to a higher risk of problematic media consumption (Kardefelt-Winther, 2014). The I-PACE model (Interaction of Person-Affect-Cognition-Execution; Brand et al., 2016, 2019) was developed as a theoretical framework to explain developments of problematic Internet usage and maintenance of specific (Internet) use disorders. It integrates personal predispositions (e.g. personality, learning experiences), affective and cognitive reactions (e.g. emotional states, usage beliefs), and executive functions (e.g. self-regulation, impulse control). These factors interact dynamically and reinforce each other so that short-term rewards (e.g. stress reduction through social media use) lead to an increased susceptibility to addictive behaviors in the long term. The model emphasizes the importance of neurobiological and psychological mechanisms in an interactive process. Within this framework, situational triggers and executive control are of particular importance. For instance, the “brain drain” effect (Ward et al., 2017) suggests that the mere presence of a smartphone occupies cognitive resources. While individual studies in this field have yielded heterogeneous results, a *meta*-analysis by Böttger et al. (2023) confirmed its overall significance, concluding that the device’s presence can indeed impair cognitive functions (but small effect size). Furthermore, research by Exelmans and Van den Bulck (2016) highlights that mobile phone use in bed after lights out is a common behavior associated with poorer sleep quality and insomnia. Such pre-sleep usage is inherently enabled by the device’s proximity in private contexts such as the bedroom, which may further impair executive control and trigger unplanned digital immersion.

Based on the aforementioned models, it becomes apparent that maladaptive patterns in the use of certain digital media can lead to the development of Internet use disorder, which shares similarities with other behavioral addictions.

For the first time, the ICD-11 (International Classification of Diseases − 11) includes a digital media-related use disorder in form of the gaming disorder diagnosis (World Health Organization, 2019). At the moment, it is discussed in how far related behaviors such as disordered social media use might also get a distinct diagnosis in the future (Brand et al., 2022, Montag and Markett, 2024). In ICD-11, gaming disorder and other specific disorders due to addictive behaviors are characterized by diagnostic requirements including impaired control over the behavior (e.g., onset, frequency, intensity, duration, termination, context); increasing priority in contrast to other life interests and daily activities; and continuation or escalation of the behavior despite negative consequences (e.g., family conflict, poor scholastic performance, negative impact on health); as well as the occurrence of functional impairment or marked distress in every-day-life (Saunders et al., 2025). For illustrations: Montag and Elhai, 2023, Montag and Markett, 2024, Montag and Rumpf, 2021 investigated in what areas functional impairments most often occurred in the context of Gaming Disorder.

Due to the negative consequences of Internet use disorder, intervention options have been developed and evidence regarding their effectiveness is increasing (Rumpf et al., 2025). These include, for example, methods based on therapeutic approaches, such as approaches from cognitive behavioral therapy (Wölfling et al., 2019, Wölfling et al., 2025), preventive approaches (Lindenberg et al., 2025) and apps or changes in smartphone settings (Greyscale Setting, App Blocker; Augner et al., 2022).

Beyond these interventions, a simple other intervention might be of help to reduce Internet use disorder, namely by improving the structure in everyday life: As Montag et al. (2015) have described, the use of the clock or the alarm function of the smartphone is one of the most undervalued functions, which might lead to a large number of daily screen activations and subsequent smartphone and Internet use. Given this high frequency (in contrast to lower-frequency functions like checking a calendar), reducing screen activations to read the time or use the smartphone as an alarm clock by using the so-called “timekeeper” might be another intervention measure to reduce problematic smartphone and Internet use. While using traditional “timekeepers” does not strictly preclude the presence of a smartphone, it facilitates a functional separation that reduces the necessity of the device in sensitive contexts. Supporting this logic, an intervention study by Hughes and Burke (2018) showed that restricting smartphone use in the bedroom, which shifts essential functions like alarm-setting to traditional alternatives, significantly decreases the risk of smartphone addiction. This underscores the potential of traditional devices to serve as protective tools. As Montag et al. (2015) stated ***“***We borrowed the term ‘zeitgeber’ from a different context – namely chronobiology (e.g. Arendt and Broadway, 1987). Here, we refer to its original German meaning – literally to ‘give or show the time’” (p. 24). The following study will therefore focus on the effect of timekeeper (zeitgeber) on problematic Internet and smartphone use.

### Hypotheses

Considering the need for replication in psychology (Pashler & Wagenmakers, 2012), attempts to replicate and extend study findings are crucial to determine how and if timekeepers might be effective in reducing Internet use disorder. Theoretically, the use of traditional timekeepers serves as a pre-emptive strategy to reduce digital triggers. Rather than “distracting” the user from the smartphone or interrupting an ongoing usage chain, these devices ensure that the initial encounter with the smartphone is prevented. By utilizing a traditional watch or alarm clock, the “gateway” to the digital environment remains closed, thereby pre-empting the situational cues that typically initiate unplanned and potentially problematic Internet use. Therefore, the following hypotheses − oriented towards the timekeeper study by Montag et al. (2015) – test these associations:

H1: People who subjectively rate their Internet use as overuse have higher rates of Internet use disorder than people who do not.

H2: People who unintentionally stay on the phone after checking the time have higher rates of Internet use disorder than people who do not.

H3: People who do not wear a watch show higher rates of Internet use disorder than people with a traditional watch, digital watch, or smartwatch.

H4: People who use a “real” alarm clock show lower rates of Internet use disorder than those who use their smartphone as an alarm clock.

H5: People who take their smartphone into the bedroom show higher rates of Internet use disorder than those who do not.

H6: People who let their smartphone wake them up in the morning show higher rates of Internet use disorder than those who do not.

H7: The time of first smartphone use after waking up and the time of last use before going to sleep "predict" Internet use disorder (not meant in a causal way here, we investigate cross-sectional data).

## 2 Method

### 2.1 Sample

The present data were collected within a smartphone application designed to reduce problematic Internet use. The project name is SCAPIT (Stepped Care Approach for Problematic Internet use Treatment) and the overall research protocol has been described elsewhere (Bischof et al., 2022). See also further information about this project in other papers which have been published relying on data related to this project (Montag et al., 2024a, Montag et al., 2024b). Unlike these previous works, which reported the study protocol (Bischof et al., 2022), investigated characteristics of e-coach users (Montag et al., 2024a), or examined COVID-19-related changes in Internet use and well-being (Montag et al., 2024b), the present study specifically examines the association between the use of timekeepers and problematic Internet use, focusing on everyday habits rather than intervention outcomes or pandemic-related effects. The study was approved by the ethics committee of the University of Lübeck.

For the present study, it is of relevance that the SCAPIT project was advertised via different media channels to promote a smartphone app aiming at achieving a healthier online behavior. Recruitment included several channels such as universities, schools, workplaces, social media, press and TV releases, paid digital adds, and paid influencers. This might have drawn a population with increased frequent and intensive use of smartphones and Internet to the app (more insights into person characteristics can be found here: Montag et al., 2024a, Montag et al., 2024b). After downloading the app and consenting to be part of the project, participants were asked to fill in a number of questionnaires including items on timekeepers.

The sample consisted of *N* = 6,631 people. Of these, *n_m_* = 3,468 (52.3%) identified themselves as male, *n*_w_ = 3,045 (45.9%) as female, and *n*_d_ = 118 (1.8%) as diverse.

The age of the participants was between 16 and 67 years, with an average age of *M_age_* = 28.30 years (*SD_a_*_ge_ = 12.67). With regard to marital status, *n* = 6,631 participants provided usable information. The largest proportion of respondents were single (*n* = 4,685; 70.6%), followed by people who were married and living with their spouse (*n* = 1,333; 20.1%) and participants who were in a registered partnership (*n* = 344; 5.2%). Further, people reported to be divorced (*n* = 175; 2.6%), being married but separated (*n* = 70; 1.1%) and widowed (*n* = 24; 0.4%). Regarding the stability of their partnership situation, *n* = 5,298 participants provided valid information: Of these, *n* = 2,027 (38.3%) were living in a stable partnership, while *n* = 3,271 (61.7%) stated that they were not in a stable relationship at the time of the survey. The majority of the sample was born in Germany (*n* = 6,021; 90.8%), while *n* = 610 (9.2%) reported to born outside Germany. After data cleaning, information from N = 6,615 people was included in the analyses (*n*_male_ = 3,460 (52.3%), *n*_female_ = 3,040 (46.0%), *n*_diverse_ = 115 (1.7%), *M*_age_ = 28.31 years (*SD*_age_ = 12.66)). Participants who did not speak German were excluded.

### 2.2 Material

The participants were asked to provide socio-demographic information and were then given questions about which device they mainly use to be online and whether they use some Internet applications more than is good for them.

Afterward, the participants completed the German translation (Gürtler et al., 2014) of the Compulsive Internet Use Scale (CIUS; Meerkerk et al., 2009). As a dimensional measure, this captures the severity of Internet use disorder with 14 items, which include the symptom areas of loss of control (items 1, 2, 5, and 9), withdrawal (item 14), coping (items 12 and 13), mental and behavioral appropriation (items 4, 6 and 7) and interpersonal and intrapersonal conflicts (items 3, 8, 10 and 11). The CIUS is answered on a five-point Likert scale (0 = “Never”, 1 = “Rarely”, 2 = "Sometimes", 3 = “Often”, 4 = “Very often”), so that total sum values between 0 and 56 can be achieved (Gürtler et al., 2014). The internal consistency for the sample used was Cronbach's α = 0.90.

The participants were furthermore asked whether they usually wear a wristwatch, use a “real” alarm clock to wake up instead of a smartphone, take their cell phone/smartphone into the bedroom, and are woken up in the morning by their cell phone/smartphone. These questions could be answered dichotomously with yes or no. In addition, they answered on a 5-point Likert scale, how often they “only” check the time on their cell phone/smartphone and then unintentionally stay on the device longer than originally planned (e.g. to check emails), how soon after waking up they usually pick up their cell phone/smartphone for the first time in the morning and go online and how shortly before going to bed they usually pick up their cell phone/smartphone for the last time to go online. These 7 items are adapted from the Montag et al. (2015) timekeeper items and can be found in Appendix A.

### 2.3 Statistical analysis

The data analysis consisted of descriptive statistics, including the report of mean values and standard deviations, as well as multivariate statistical methods such as hierarchical multiple linear regressions and correlations. Since the proclaimed hypothesis are tested in the correlation analysis and checked again in the regression model, only those variables who are significant in both analyses will be deemed enough to fulfill the hypothesis. The correlation analysis was conducted using linear correlation (Pearson) between continuous variables, point biseral correlation for correlating one dichotomous variable with one continuous variable and lastly the phi-association-coefficient to test the strength of association between two dichotomous variables. The hierarchical regression model was constructed in four steps to assess the incremental validity of specific usage habits. First, sociodemographics were included to control for general background variables. In the second step, subjective overuse was added to the model to account for participants’ self-perception of problematic use, ensuring that subsequent effects reflect specific habits rather than general awareness. In step three, the dichotomous timekeeper-variables were added to assess the influence of general usage patterns and habits (e.g., device integration into the bedroom routine). Finally, in step four, the minute-based questions for smartphone usage were added to test whether quantified temporal metrics explain additional variance beyond the general usage style.

## 3 Results

Of *N* = 6,615 participants, 76.4% said they mainly use a smartphone to access the Internet. 73.2% of participants admitted that they use some Internet applications more than is good for them. 46.3% reported wearing a wristwatch (16.1% traditional, 4.1% digital, 26.1% smartwatch). With regard to their wake-up routine, 77.5% said they used their smartphone as an alarm clock. In addition, 89.5% stated that they take their smartphone into the bedroom at night. When asked about unintentional prolonged use after just checking the time on their smartphone, 29.9% said they did this “very often” or “often”. 72.9% reported using their smartphone within the first 15 min of waking up and 70.7% reported using their smartphone within the last 15 min before going to sleep. Age correlated with most of the variables examined, so age is included as a covariate in the analyses. The separate analysis of the male and female subsamples led to comparable results, which is why only the main results are reported here.

In Table 1, the bivariate correlations between all variables used can be seen for the entire data set. As expected, Internet use disorder showed strong positive correlations with subjective overuse and the tendency to remain online after checking the time on the smartphone. Negative associations were observed with the use of timekeepers such as wristwatches or real alarm clocks, indicating that individuals relying on these devices report lower levels of Internet use disorder. Furthermore, younger age was consistently related to higher CIUS scores and increased Internet use disorder, underlining age as an important covariate in subsequent analyses.

**Table 1:** Association between measured items.

	1	2	3	4	5	6	7	8	9
Compulsive Internet Use									
Subjective overuse^a^	0.499**								
Wearing a wristwatch^a^	−0.131**	−0.041**							
Using a real alarm clock^a^	−0.119**	−0.048**	0.074**						
Taking the smartphone in the bedroom^a^	0.148**	0.109**	−0.087**	−0.435**					
Waking up with the smartphone^a^	0.120**	0.063**	−0.072**	−0.709**	0.537**				
Use after-time check^b^	0.461**	0.284**	−0.086**	−0.126**	0.141**	0.150**			
Use after waking up^c^	−0.225**	−0.177**	0.028*	0.170**	−0.222**	−0.177**	−0.212**		
Use before sleeping^c^	−0.168**	−0.159**	0.028*	0.149**	−0.224**	−0.163**	−0.127**	0.374**	
Age	−0.279**	−0.121**	0.189**	0.181**	−0.295**	−0.196**	−0.122**	0.110**	0.104**

Note. n = 6615. ** p < 0.010. * p < 0.050. a = No vs Yes. b = Scale from 1 (never) to 5 (always). c = Measured in Minutes. Cronbach's Alpha of CIUS = 0.90. Association between dichotomous variables measured with Phi, between dichotomous and continous variables measured with point biseral correlation, and between two continous variables Pearson correlation is used.

The hierarchical regression analysis (Table 2) revealed that sociodemographic variables accounted for approximately 9% of the variance in Internet use disorder (Model 1). Adding subjective overuse (Model 2) substantially increased explained variance to 30%, highlighting the strong predictive value of self-perceived overuse. Incorporating timekeeper-related variables (Model 3) led to another significant improvement in model fit (ΔR^2^ = 0.10), indicating that everyday structuring habits meaningfully contribute to Internet use disorder, beyond the effect of age and gender. In the final model (Model 4), time-related variables only added marginal explanatory power (ΔR^2^ = 0.005). Across all steps, subjective overuse and the tendency to stay on the phone after checking the time emerged as the strongest predictors, even when controlling for demographics and other timekeeper behaviors.

**Table 2:** Hierarchical Regression Models for Internet Use Disorder.

	Model							
Variable	Sociodemographic		Subjective Overuse		Usage markers		Time markers	
Sex [Female]	0.088	**	0.057	**	0.008		0.011	
Age	−0.282	**	−0.225	**	−0.182	**	−0.180	**
Subjective overuse^a^			0.469	**	0.383	**	0.372	**
Wearing a wristwatch^a^					−0.052	**	−0.053	**
Using a real alarm clock^a^					−0.036	**	−0.031	**
Taking the smartphone in the bedroom^a^					−0.005		−0.019	*
Waking up with the smartphone^a^					−0.016		−0.018	
Use after-time check^b^					0.322	**	0.311	**
Use after waking up^c^							−0.064	**
Use before sleeping^c^							−0.027	*
*R*2	0.086		0.301		0.400		0.405	
Adj. *R*2	0.085		0.301		0.399		0.404	
Delta *R*2	0.086		0.216		0.098		0.005	
*F*-Change	309.975	**	2039.647	**	216.646	**	29.629	**
*N*	6615		6615		6615		6615	

Note. a = No vs Yes. b = Scale from 1 (never) to 5 (always). c = Measured in Minutes. Max VIF = 2.050. ** p < 0.010. * p < 0.050.

All regression assumptions were met. The maximum variance inflation factor (VIF = 2.04) indicated no issues of multicollinearity among predictors, ensuring that the observed effects can be interpreted as independent contributions to Internet use disorder.

**Fig. 1:**
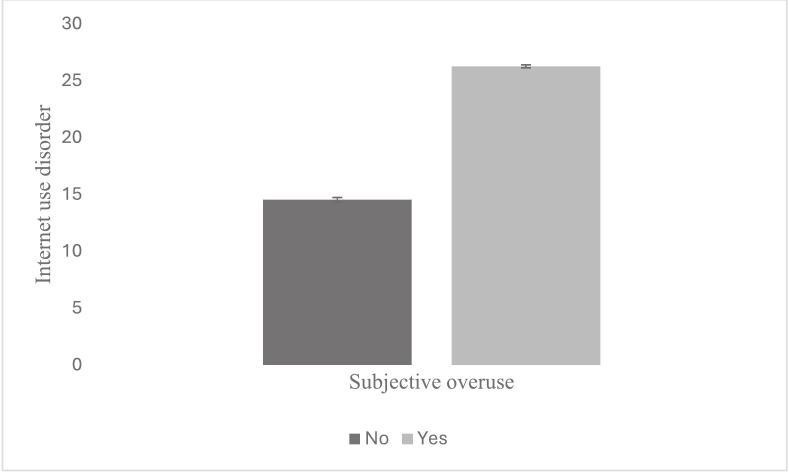
Subjective overuse is associated with a higher Internet use disorder.

**Fig. 2:**
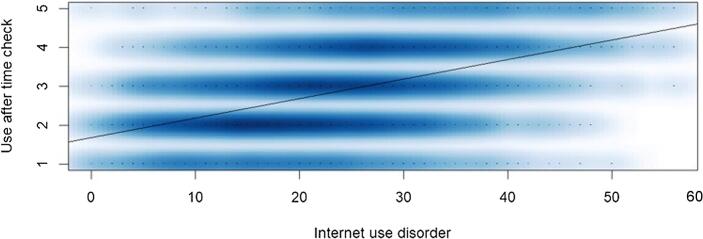
Internet use disorder is associated with use after time check (r = .46, p < .001); the time check variable ranged from 1 = never to 5 = very often (see also Appendix A).

After analyzing the correlation and regression results depicted in Table 1, Table 2 mainly two variables seem to have the highest association with Internet use disorder, namely the subjective overuse (H1, *r* = 0.499, Beta in main model = 0.372, *p* < 0.001) and the use after time-check (H2, *r* = 0.461, Beta in main model = 0.311, *p* < 0.001). It shows that a subjective overuse is associated with a higher Internet use disorder (Fig. 1) and that the higher the Internet use disorder, the greater the likelihood that someone will use their smartphone after a simple time check (Fig. 2).

All other assumed associations between CIUS and timekeeper-variables (H3-H7) were significant in the correlation analysis but not in the hierarchical regression analysis – therefore these variables are not explored further.

To explore whether specific aspects of Internet use disorder were differentially affected by timekeeper variables, separate regression models were calculated for each CIUS subscale. The full results are detailed in Table 3.

**Table 3:** Regression Models for Subscales of Compulsive Internet Use Scale.

	Subscale of CIUS									
Variable	Loss of Control		Withdrawal		Coping		Behavioral Appropriation		Inter- & Intrapersonal Conflicts	
Sex [Female]	0.024	*	0.003		0.109	**	−0.044	**	−0.036	**
Age	**−0.127**	**	**−0.106**	**	**−0.249**	**	**−0.107**	**	**−0.144**	**
Subjective overuse^a^	**0.402**	**	**0.189**	**	**0.240**	**	**0.207**	**	**0.362**	**
Wearing a wristwatch^a^	−0.027	**	−0.042	**	−0.050	**	−0.071	**	−0.037	**
Using a real alarm clock^a^	−0.001		−0.024		−0.038	*	−0.055	**	−0.021	
Taking the smartphone in the bedroom^a^	−0.014		−0.033	*	0.033	*	−0.021		−0.040	**
Waking up with the smartphone^a^	0.012		−0.014		0.002		−0.039	*	−0.037	*
Use after-time check^b^	**0.294**	**	**0.284**	**	**0.196**	**	**0.227**	**	**0.271**	**
Use after waking up^c^	−0.058	**	−0.044	**	−0.052	**	−0.056	**	−0.047	**
Use before sleeping^c^	−0.057	**	0.026	*	−0.031	**	0.010		−0.017	
*R^2^*	0.400		0.180		0.284		0.165		0.314	
*Adj. R^2^*	3.999		0.178		0.283		0.164		0.313	
*F*	439.596	**	144.481	**	262.179	**	130.680	**	302.920	**
*N*	6615		6615		6615		6615		6615	

Note. a = No vs Yes. b = Scale from 1 (never) to 5 (always). c = Measured in Minutes. ** p < 0.010. * p < 0.050.

The analyses show that the “loss of control” and “interpersonal and intrapersonal conflicts” dimensions were most strongly associated with subjective overuse and post-time-check behavior. Wearing a wristwatch and using a real alarm clock were negatively associated with several subscales, particularly “behavioral appropriation” and “coping”, suggesting that structured routines and reduced smartphone availability mitigate compulsive tendencies. In contrast, the timing variables (use after waking up / before sleeping) had only small and inconsistent effects. These results confirm that habitual patterns surrounding smartphone-as-timekeeper use are linked primarily to impulsive and self-regulatory components of Internet use disorder.

## 4 Discussion

This study investigated whether the use of timekeepers − such as wristwatches or classic alarm clocks − is associated with lower Internet use disorder. It was found that subjective overuse is associated with a higher Internet use disorder and that the higher the Internet use disorder, the greater the likelihood that someone will use their smartphone after a simple time check (Fig. 2). The results of this study therefore might indicate that avoiding the smartphone as a timekeeper is associated with a lower risk of drifting into problematic online behavior. However, due to the cross-sectional nature of the study, no causal conclusions can be drawn regarding the direction of the effect. While the theoretical model assumes that traditional timekeepers prevent usage chains by reducing digital triggers, it is also plausible that individuals with lower Internet use disorder are simply more inclined to use devices in the first place. This said, the time check item was formulated in a directed way, namely that checking the time resulted in prolonged smartphone use.

Overall, these results are in line with a previous study on using the smartphone as a timekeeper and problematic Internet use (Montag et al. (2015). The present study replicates and extends these findings in a larger sample. In addition, according to Montag et al. (2015), the smartphone might act as a gatekeeper for unplanned Internet use: the simple time display on the smartphone turns out to be a temptation to draw users into other apps or activities. Notably, this mechanism remains relevant even with modern device features like “always-on” displays or “tap to wake” functions that allow checking the time without fully unlocking the phone. Even these brief interactions might serve as cues, as notification icons on the lock screen may still trigger the urge to unlock the device and engage in further Internet usage. This underlines how detrimental the lack of separation between the pure clock function and the many other services offered by the smartphone might be. The smartphone offers multifunctional all-in-one access − it satisfies the need for information, social interaction, entertainment, and also the need to keep an eye on the time. A traditional alarm clock or watch, on the other hand, only satisfies the need for time and offers no other temptations. The finding that traditional timers go hand in hand with less Internet use thus reflects the idea that fewer multifunctional stimuli in everyday life lead to less unplanned media consumption. Intriguingly, we could also observe rising CIUS scores from wearing a traditional wristwatch over a digital wristwatch over a smartwatch to not wearing a watch at all (the latter group likely relies on a smartphone as a timekeeper). Hence, also wearing the Internet connected smartwatch might pull people into excessive use of digital functions beyond the clock, because of incoming notifications. The individual CIUS values for the groups are as follows: *M_traditional_* = 20.58 (*SD_traditional_* = 10.15, *N* = 1,062), *M_digital_* = 22.47 (*SD_digital_* = 10.77, *N* = 274), *M_smartwatch_* = 22.17 (*SD_smartwatch_* = 10.27, *N* = 1,727) and *M_none_* = 24.38 (*SD_none_* = 10.31, *N* = 3,552).

The I-PACE model (Brand et al., 2016, Brand et al., 2019) can be used to explain the results. The constant presence of the smartphone and its use as a timekeeper represent an almost omnipresent situational trigger. Accordingly, it can be seen that technological triggers − such as notification sounds, screens that light up or routinely reaching for a mobile phone – might encourage the development of problematic or even addictive patterns of use. If the smartphone is constantly carried around and used at every opportunity, this is likely to reinforce attention bias and automatic behavior. The result that many participants “get lost” when checking the time suggests that executive control (in the sense of the I-PACE “execution” aspect) fails in such situations: although the user only intends to use the device briefly (checking the time), they then fall into habitual usage loops, for example, because incoming messages or available content trigger automated impulses to act.

Despite the findings, some methodological limitations of the study must be taken into account. Firstly, it is a cross-sectional research design, which means that causal conclusions are only possible to a limited extent (here by asking if the phone behavior is prolonged after checking the time on the phone). Secondly, the data is based exclusively on self-reports by the participants. Such self-reported measures are susceptible to various distortions. For example, participants tend not to accurately assess their own Internet and smartphone usage. Previous work shows that the actual usage time often deviates from the self-assessed values − people often tend to underestimate their consumption (Parry et al., 2021, Wickord and Quaiser-Pohl, 2022). Moreover, objectively-measured and self-reported digital technology use may be differently related to psychological outcomes (Rozgonjuk, et al., 2021). Thirdly, the age distribution of the sample must be taken into account. Our participants are younger compared to a general population sample. However, age is an important factor, especially in the context of the question examined here: younger people use smartphones more frequently and have grown up with digital technologies as digital natives, while older people, the so-called digital immigrants, tend to stick to more traditional habits (such as wearing wristwatches). Finally, it should be noted that other confounding factors that were not surveyed may have played a role (see potential bias due to recruitment as described in the methods): For example, personality traits could influence both the choice of timekeeper and Internet use.

Despite the limitations mentioned, the results open up important perspectives for future research. Experimental designs could make additional valuable contributions. Methodologically, future studies should make greater use of objective measurement methods. For example, app trackers could be installed on participants' smartphones to record exactly when and for how long the device is used and even recognize specific behavioral patterns (e.g. unlocking the phone just to read the time vs. staying on it for longer). Such objective digital data would paint a much more accurate picture of usage behavior, e.g., to conduct diary studies or experience sampling, in which participants regularly provide feedback on their current state of health and usage behavior via apps. The use of smartphone sensors, log files, and machine learning analyses would make it possible to determine more precisely the role of traditional vs. digital timekeepers in everyday routines in the development of Internet use disorder. Hence, mobile sensing and digital phenotyping approaches will be helpful to make progress in the present research field (Montag & Rumpf, 2021).

In addition to these research implications, practical recommendations might be derived. The results could suggest that even simple changes in everyday behavior can have a preventive effect. For example, users with excessive smartphone or Internet use could be advised to consciously acquire traditional timer keepers again and set up certain mobile phone-free zones (bedroom) or times (after waking up) in order to reduce their Internet use disorder. As already postulated by Montag et al. (2015), it is essential to separate traditional and digital spheres in everyday life again to prevent excessive media use. Future research on preventative measures should therefore aim at investigating if promoting the use of traditional timekeepers can help to regain the time that is at risk of being lost in the maelstrom of digital devices (and the platforms operating on them with a data business model; Montag & Elhai, 2023). Further, future research endeavors should be more fine-granular regarding the diagnostics of excessive online use. It will be interesting which online-use-domains will be most closely related to not using traditional timekeepers, is it social media, video games, e-mails or something different (Montag et al., 2021)?

## Funding

The present project belongs to the SCAPIT study, which has been funded by the Innovation Fund of the Federal Joint Committee Germany (Grant number 01NVF19031).

## CRediT authorship contribution statement

**Lea-Christin Wickord:** Writing – review & editing, Writing – original draft, Visualization, Validation. **Anja Bischof:** Writing – review & editing, Conceptualization. **Christopher Kannen:** Writing – review & editing, Conceptualization. **Dimitri Löchner:** Writing – review & editing, Conceptualization. **Harriet Salbach:** Writing – review & editing, Conceptualization. **Thomas Mößle:** Writing – review & editing, Conceptualization. **Lara Zumrode:** Writing – review & editing, Conceptualization. **Klaus Wölfling:** Writing – review & editing, Conceptualization. **Hans-Jürgen Rumpf:** Writing – review & editing, Conceptualization. **Christian Montag:** Writing – review & editing, Conceptualization.

## Declaration of competing interest

The authors declare that they have no known competing financial interests or personal relationships that could have appeared to influence the work reported in this paper.

## Figures and Tables

**Table:** 

Ich trage meistens eine Armbanduhr. (I usually wear a wristwatch.)	Ja, eine analoge Armbanduhr/ Yes, a traditional wristwatch Ja, eine digitale Armbanduhr/ Yes, a digital wristwatch Ja, eine Smartwatch/ Yes, a smartwatch Nein/ No	
Ich nutze einen „echten“ Wecker zum Wecken, anstatt mein Mobiltelefon/Smartphone dafür zu verwenden. (I use an ‘actual’ alarm clock to wake me up instead of using my mobile-/ smartphone.)	Ja/Yes	Nein/No
Ich nehme mein Mobiltelefon / Smartphone mit in mein Schlafzimmer. (I take my mobile-/ smartphone into my bedroom.)	Ja/Yes	Nein/No
Ich lasse mich morgens durch mein Mobiltelefon / Smartphone wecken. (I let my mobile-/ smartphone wake me up in the morning.)	Ja/Yes	Nein/No

## Data Availability

Data will be made available on request.

## Appendix

### Appendix A

Timekeeper Items.

Smartphone/Mobile phone use as a 'timekeeper' (showing the time) in daily life.

For research purposes we are interested in your answers to the following questions. Please indicate the applicable answers.Wie häufig wollen Sie „nur“ die Uhrzeit auf dem Mobiltelefon / Smartphone nachschauen und bleiben danach trotzdem unbeabsichtigt länger am Gerät als ursprünglich geplant (z.B. zum Checken von E-Mails)? (How often do you “just” want to check the time on your mobile-/ smartphone and then unintentionally stay on the device longer than originally planned (e.g. to check emails)?).

Antwort: Nie − Selten − Manchmal − Oft − Sehr oft (Answer: Never – seldom – sometimes – often – very often).

Wie schnell nach dem Aufwachen nehmen Sie üblicherweise morgens Ihr Mobiltelefon / Smartphone zum ersten Mal in die Hand und gehen online? (How soon after waking up do you usually pick up your mobile-/ smartphone for the first time in the morning and go online?).

Antwort: Minuten oder mehr als 60 Minuten (Answer: Minutes or more than 60 min).

Wie kurz vor dem Schlafengehen nehmen Sie üblicherweise Ihr Mobiltelefon / Smartphone zum letzten Mal in die Hand, um online zu gehen? (How close to bedtime do you usually pick up your mobile-/ smartphone for the last time to go online?).

Antwort: Minuten oder mehr als 60 Minuten (Answer: Minutes or more than 60 min).

## References

[b0005] Augner C., Vlasak T., Aichhorn W., Barth A. (2022). Tackling the ‘digital pandemic’: The effectiveness of psychological intervention strategies in problematic internet and smartphone use—A meta-analysis. Australian & New Zealand Journal of Psychiatry.

[b0010] Bischof A., Brandt D., Schlossarek S., Vens M., Rozgonjuk D., Wernicke J., Kannen C., Wölfling K., Dreier M., Salbach H., Basenach L., Mößle T., Olbrich D., König I., Borgwardt S., Montag C., Rumpf H.-J. (2022). Study protocol for a randomised controlled trial of an e-health stepped care approach for the treatment of internet use disorders versus a placebo condition: The SCAPIT study. BMJ Open.

[b0015] Böttger T., Poschik M., Zierer K. (2023). Does the brain drain effect really exist?. A meta-analysis. Behavioral Sciences.

[b0020] Brand M., Rumpf H.J., Demetrovics Z., MÜller A., Stark R., King D.L., Potenza M.N. (2022). Which conditions should be considered as disorders in the International Classification of Diseases (ICD-11) designation of “other specified disorders due to addictive behaviors”?. Journal of behavioral addictions.

[b0030] Brand M., Wegmann E., Stark R., Müller A., Wölfling K., Robbins T.W., Potenza M.N. (2019). The Interaction of Person-Affect-Cognition-Execution (I-PACE) model for addictive behaviors: Update, generalization to addictive behaviors beyond Internet-use disorders, and specification of the process character of addictive behaviors. Neuroscience & Biobehavioral Reviews.

[b0035] Brand M., Young K.S., Laier C., Wölfling K., Potenza M.N. (2016). Integrating psychological and neurobiological considerations regarding the development and maintenance of specific Internet-use disorders: An Interaction of Person-Affect Cognition-Execution (I-PACE) model. Neuroscience & Biobehavioral Reviews.

[b0040] Elhai J.D., Dvorak R.D., Levine J.C., Hall B.J. (2017). Problematic smartphone use: A conceptual overview and systematic review of relations with anxiety and depression psychopathology. Journal of Affective Disorders.

[b0045] Exelmans L., Van Den Bulck J. (2016). Bedtime mobile phone use and sleep in adults. Social Science & Medicine.

[b0050] Gürtler D., Rumpf H.-J., Bischof A., Kastirke N., Meerkerk G.-J., John U., Meyer C. (2014). Psychometrische Eigenschaften und Normierung der deutschen Version der Compulsive Internet Use Scale (CIUS) [Psychometric properties and standardization of the German version of the Compulsive Internet Use Scale (CIUS)]. Diagnostica.

[b0055] Hughes N., Burke J. (2018). Sleeping with the frenemy: How restricting ‘bedroom use’ of smartphones impacts happiness and wellbeing. Computers in Human Behavior.

[b0060] Kardefelt-Winther D. (2014). A conceptual and methodological critique of internet addiction research: Towards a model of compensatory internet use. Computers in Human Behavior.

[b0065] Kuss, D. J., & Pontes, H. M. (2019). *Internet addiction.* (Vol. 41). Hogrefe Publishing. Doi: 10.1027/00501-000.

[b0070] Lindenberg K., Petersen K.U., Brandhorst I., Müller K.W., Spahn M., Wölfling K., Wartberg L., Wirtz M., Bischof A., Rumpf H.-J. (2025). Guideline on early interventions for internet use disorders. *SUCHT – Interdisciplinary Journal of*. Addiction Research.

[bib191] Mangold R., Vorderer P., Bente G. (2004). *Lehrbuch der Medienpsychologie*.

[b0075] Meerkerk G.-J., Van den Eijnden R.J.J.M., Vermulst A.A., Garretsen H.F.L. (2009). The Compulsive Internet Use Scale (CIUS): Some psychometric properties. CyberPsychology & Behavior.

[b0085] Montag C., Elhai J.D. (2023). On social media design, (online-)time well-spent and addictive behaviors in the age of surveillance capitalism. Current Addiction Reports.

[b0090] Montag C., Elhai J.D., Kannen C., Bischof A., Brandt D., Schmidt H., Rozgonjuk D., Rumpf H.-J. (2024). Insights into psychological characteristics of persons (not) agreeing to use an e-coach-application to reduce elevated internet Use Disorder tendencies. Addictive Behaviors Reports.

[b0095] Montag C., Kannen C., Lachmann B., Sariyska R., Duke É., Reuter M., Markowetz A. (2015). The importance of analogue zeitgebers to reduce digital addictive tendencies in the 21st century. Addictive Behaviors Reports.

[b0100] Montag C., Markett S. (2024). Depressive inclinations mediate the association between personality (neuroticism/conscientiousness) and TikTok Use Disorder tendencies. BMC Psychology.

[b0105] Montag C., Pontes H.M., Kannen C., Rozgonjuk D., Brandt D., Bischof A., Salbach H., Mößle T., Wölfling K., Rumpf H.-J. (2024). Examining the interplay between internet use disorder tendencies and well-being in relation to sofalizing during the COVID-19 pandemic. Comprehensive Psychiatry.

[b0110] Montag C., Rumpf H.-J. (2021). The potential of digital phenotyping and mobile sensing for psycho-diagnostics of internet use disorders. Current Addiction Reports.

[b0115] Montag C., Wegmann E., Sariyska R., Demetrovics Z., Brand M. (2021). How to overcome taxonomical problems in the study of internet use disorders and what to do with “smartphone addiction”?. Journal of Behavioral Addictions.

[b0120] Panova T., Carbonell X. (2018). Is smartphone addiction really an addiction?. Journal of Behavioral Addictions.

[b0125] Parry D.A., Davidson B.I., Sewall C.J.R., Fisher J.T., Mieczkowski H., Quintana D.S. (2021). A systematic review and meta-analysis of discrepancies between logged and self-reported digital media use. Nature Human Behaviour.

[b0130] Pashler H., Wagenmakers E.-J. (2012). Editors’ introduction to the special section on replicability in psychological science: A crisis of confidence?. Perspectives on Psychological Science.

[b0135] Rozgonjuk D., Elhai J.D., Sapci O., Montag C. (2021). Discrepancies between self-reports and behavior: Fear of Missing out (FoMO), self-reported problematic smartphone use severity, and objectively measured smartphone use. Digital Psychology.

[b0145] Rumpf H.-J., Batra A., Hoch E., Mann K., Thomasius R., Bischof A., Brand M. (2025). Guidelines on internet use disorders: Introduction and methodology. *SUCHT – Interdisciplinary Journal of*. Addiction Research.

[b0150] Saunders J.B., Rumpf H.-J., Carragher N., Poznyak V. (2025). The development of and rationale for gaming disorder in ICD-11 and a review of available assessment tools. Current Addiction Reports.

[b0155] Statista. (2024, November 19). *Anzahl der Smartphone-Nutzer in Deutschland bis 2030* [Number of smartphone users in Germany until 2030]. https://de.statista.com/statistik/daten/studie/198959/umfrage/anzahl-der-smartphonenutzer-in-deutschland-seit-2010/.

[b0160] Ward A.F., Duke K., Gneezy A., Bos M.W. (2017). Brain drain: The mere presence of one’s own smartphone reduces available cognitive capacity. Journal of the Association for Consumer Research.

[b0165] Whiting A., Williams D. (2013). Why people use social media: A uses and gratifications approach. Qualitative Market Research: An International Journal.

[b0170] Wickord L.-C., Quaiser-Pohl C. (2023). Suffering from problematic smartphone use? why not use grayscale setting as an intervention!–an experimental study. Computers in Human Behavior Reports.

[b0175] Wickord L.-C., Quaiser-Pohl C.M. (2022). Does the type of smartphone usage behavior influence problematic smartphone use and the related stress perception?. Behavioral Sciences.

[b0180] World Health Organization. (2019). Disorders due to addictive behaviours. In *ICD-11 for Mortality and Morbidity Statistics*. Retrieved February 6, 2026, from https://icd.who.int/browse/2026-01/mms/en#499894965.

[b0185] Wölfling K., Eichenberg C., Leménager T., Basenach L., Dreier M., Rüther T., Salbach H., Bischof A., Rumpf H.-J. (2025). Guideline on the treatment of general internet use disorder. *SUCHT – Interdisciplinary Journal of*. Addiction Research.

[b0190] Wölfling K., Müller K.W., Dreier M., Ruckes C., Deuster O., Batra A., Mann K., Musalek M., Schuster A., Lemenager T., Hanke S., Beutel M.E. (2019). Efficacy of short-term treatment of internet and computer game addiction: A randomized clinical trial. JAMA Psychiatry.

